# Response to: The BRAIN TRIAL: a randomised, placebo controlled trial of a Bradykinin B2 receptor antagonist (Anatibant) in patients with traumatic brain injury

**DOI:** 10.1186/1745-6215-10-110

**Published:** 2009-12-03

**Authors:** Vincent F Simmon

**Affiliations:** 1 Xytis, Inc, 101 Theory, Suite 100, Irvine, CA 92617, USA

## Abstract

A response to and comment on **The BRAIN TRIAL: a randomised, placebo controlled trial of a Bradykinin B2 receptor antagonist (Anatibant) in patients with traumatic brain injury**, by Haleema Shakur, Ian Roberts, et al.

## Letter

### Ommisions and inaccuracies

As stated in the BRAIN TRIAL manuscript [1], Xytis was the financial Sponsor of this clinical trial, during the course of which there were substantive disagreements between the Sponsor and LSHTM. As pointed out in the manuscript, the Trial was interrupted due to safety concerns on 1 November, 2007 based on an un-blinded analysis of the data to that point by the DSMB.

The BRAIN TRIAL manuscript clearly states the definition of a Serious Adverse Event. What is not said is that in this clinical trial, LSHTM used **two **versions of SAEs. The first version was a completed CRF (absent an SAE form) in which the investigator ticked the box indicating that an adverse event was serious, but did not fill out the SAE form, which is a regulatory requirement for an SAE. A separate entity, HPM, had responsibility for reporting SAEs (that is the real SAEs on SAE forms) to Xytis and to the appropriate regulatory authorities. Investigators ticked about twice as many Adverse Events, Serious boxes as they filled out SAE forms. While the BRAIN TRIAL manuscript treats SAEs as always being a unique population, in fact the reconciliation of Adverse Events, Serious with real SAEs reported to HPM was not accomplished until May of 2008, despite the fact that LSHTM received the SAE forms from the investigators and forwarded them to HPM. As of 1 November, fully 56.4% (54 out of 94) of the Adverse Events, Serious reported to the DSMB were actually adverse events that were not Serious Adverse Events according to the protocol (as subsequently reported by LSHTM). In fact, at the time the trial was stopped, HPM had received only 49 SAEs, and as required, had reported these to regulatory authorities. Thus the decision by the DSMB to interrupt the trial was based on mostly (56.4%) erroneous information. The failure of LSHTM to "clean" the database in a timely manner was a direct cause of the excessive accumulation of Adverse Events, Serious.

The Manuscript inaccurately states the Study Objectives, which were defined by the protocol as a Primary Objective "to evaluate the safety of different doses of XY2405 when used as a treatment for acute TBI in order to inform dose selection for a phase III trial" and a Secondary Objective "to assess the effect of XY2405 on mortality, morbidity among patients with acute TBI".

Xytis' concern is that the BRAIN TRIAL manuscript is biased. For example while the authors performed both Per Protocol and Intent to Treat analyses, the authors selectively chose data from one or the other for presentation.

Another example of bias is when the authors state "It is unknown to the authors whether the decision to withdraw funding was influenced by the unscheduled un-blinded analyses", is patently false. It is well known by the LSHTM authors that Xytis formally requested termination of the trial in mid-November, long before Xytis had access to un-blinded data.

In terms of un-blinding, it should be noted that I was the only Xytis employee who had access to un-blinded data, and I was not involved in the conduct of the trial or the analysis of the data, which was under the control of LSHTM. However, several LSHTM employees who had direct involvement in the trial (Haleema Shakur and Ian Roberts) reviewed un-blinded data while queries were ongoing and prior to the final data analysis. The impact of these un-blindings on their analysis is not known.

The HIREOS scale has not been published, and as such may be a source of bias.

### Additional Analysis of the Data from the BRAIN TRIAL

Table 1 below indicates the deaths of patients versus their initial or entry GCS score and their treatment (placebo, low, medium or high dose XY2405).

**Table 1:** GCS score compared with the number of deaths in each treatment group, as well as the total deaths for each GCS group.

GCS	Placebo	Low	Medium	High	Total Deaths	Deaths/patient treated by cohort
3	3	0	0	3	6	16%
	
4	0	0	0	1	1	
	
5	1	1	1	0	3	

6	2	7	3	0	12	32%
	
7	0	1	3	2	6	
	
8	3	0	1	0	4	

9	2	0	2	2	6	15%
	
10	0	0	1	1	2	
	
11	0	0	0	0	2	
	
12	0	1	1	1	3	

Total Deaths	11	10	12	12	45	

The three cohorts (GCS 3-5, GCS 6-8, and GCS 9-12) are the same cohorts used in the BRAIN TRIAL to achieve balance among treatment groups.

It is apparent that deaths are about even among the four treatment arms. What is striking however, is the death rate among the three GCS cohorts. One would have expected that patients with GCS scores of 3, 4 and 5 would have suffered the highest death rate. The average of this group was 16% mortality. The average of the next highest cohort of GCS entries (GCS 6 - 8) was 32%, twice that of the supposedly more severe traumatic brain injury cohort. This is a most unexpected result. A careful review of the protocol as well as the training instructions given to investigators indicates that no policy or procedures were established to assure that a consistent initial GCS was taken. The nature of TBI is that many patients, particularly those with polytrauma, are partially or completely sedated, on ventilators, etc., and assessment of their initial GCS score can vary considerably over a few hours. Strict criteria are needed to ensure that all clinical sites use the same criteria for the entry GCS score. The data from the BRAIN TRIAL clearly indicates that the initial GCS score is not reliable in this trial. Inasmuch as the authors "adjusted" the data, including the DRS and HIREOS, to take into account the entry GCS scores, their entire analysis is flawed.

In my opinion, the BRAIN TRIAL manuscript is both biased and flawed, and cannot be relied upon as a reliable presentation of the data from this clinical trial.

## Abbreviations

DRS: Disability Rating Scale; DSMB: Data Safety Monitoring Board; GCS: Glasgow Coma Scale; HIREOS: Head Injury Related Early Outcome Score; LSHTM: London School of Hygiene and Tropical Medicine; NIH: National Institutes of Health; HPM: Healthcare Project Management, Geneva; CRF: Case report form.

## Competing interests

Xytis and LSHTM had an adversarial relationship, resulting in legal proceedings in the High Court in London. The potential that bias may exist in this Letter, as well as in the BRAIN TRIAL manuscript should be considered.

## Disclaimer

The factual contents of this paper and the details provided about the clinical trial reported therein have not been verified by BioMed Central Limited ('the publisher') its agents or employees. This article has been published in accordance with this journal's obligation to report all clinical trials, in the public interest. No liability is accepted by the publisher, its agents or employees in connection with the contents of this paper.

## Author's information

Dr. Simmon has been the CEO and President, and COO of a number of public and private biotechnology companies, including Alpha 1 Biomedicals, Cortex Pharmaceuticals, Merrimack Pharmaceuticals, and Xytis, Inc. In those capacities, he has been responsible for the conduct of Phase II clinical trials evaluating new therapeutic agents for Hepatitis B, Hepatitis C, Schizophrenia, Alzheimer's disease, Mild Cognitive Impairment, Rheumatoid Arthritis, Psoriasis, AIDS and an AIDS vaccine. Among the academic and research institutions involved in these clinical studies were NIH, Harvard Medical School, Stanford University, University of California San Francisco, University of California Los Angeles, Wayne State University, and the University of Miami. He has authored or co-authored more than 30 peer reviewed scientific articles.
